# Cardiac MR Elastography: Comparison with left ventricular pressure measurement

**DOI:** 10.1186/1532-429X-11-44

**Published:** 2009-11-09

**Authors:** Thomas Elgeti, Michael Laule, Nikola Kaufels, Jörg Schnorr, Bernd Hamm, Abbas Samani, Jürgen Braun, Ingolf Sack

**Affiliations:** 1 Department of Radiology, Charité - Universitätsmedizin Berlin, Campus Mitte, Charitéplatz 1, 10117 Berlin, Germany; 2 Department of Medicine (Cardiology, Angiology, Pulmonology) Charité - Universitätsmedizin Berlin, Campus Mitte, Berlin, Germany; 3 Department of Medical Biophysics, University of Western Ontario, Ontario, Canada; 4 Department of Electrical and Computer Engineering, University of Western Ontario, Ontario, Canada; 5 Institute of Medical Informatics, Charité - Universitätsmedizin Berlin, Campus Benjamin Franklin, Hindenburgdamm 30, 12200 Berlin, Germany

## Abstract

**Purpose of study:**

To compare magnetic resonance elastography (MRE) with ventricular pressure changes in an animal model.

**Methods:**

Three pigs of different cardiac physiology (weight, 25 to 53 kg; heart rate, 61 to 93 bpm; left ventricular [LV] end-diastolic volume, 35 to 70 ml) were subjected to invasive LV pressure measurement by catheter and noninvasive cardiac MRE. Cardiac MRE was performed in a short-axis view of the heart and applying a 48.3-Hz shear-wave stimulus. Relative changes in LV-shear wave amplitudes during the cardiac cycle were analyzed. Correlation coefficients between wave amplitudes and LV pressure as well as between wave amplitudes and LV diameter were determined.

**Results:**

A relationship between MRE and LV pressure was observed in all three animals (R^2 ^≥ 0.76). No correlation was observed between MRE and LV diameter (R^2 ^≤ 0.15). Instead, shear wave amplitudes decreased 102 ± 58 ms earlier than LV diameters at systole and amplitudes increased 175 ± 40 ms before LV dilatation at diastole. Amplitude ratios between diastole and systole ranged from 2.0 to 2.8, corresponding to LV pressure differences of 60 to 73 mmHg.

**Conclusion:**

Externally induced shear waves provide information reflecting intraventricular pressure changes which, if substantiated in further experiments, has potential to make cardiac MRE a unique noninvasive imaging modality for measuring pressure-volume function of the heart.

## Introduction

Today several cardiac imaging modalities are available that provide excellent information on cardiac morphology and tissue structure with high temporal resolution [1-4]. However, as these modalities cannot measure forces, insight into myocardial physiology, particularly initiation of contraction and relaxation, is limited. To obtain information on cardiac elasticity and contractility in a clinical setting, it is still necessary to perform invasive catheterization for direct measurement of pressure in the chamber of interest [5]. Such information is very important as abnormal alteration of ventricular pressure between systole and diastole can sensitively indicate a variety of diseases such as hypertrophy or dysfunctional diastolic relaxation [5-7].

Recently cardiac magnetic resonance elastography (MRE) [8] was introduced as a noninvasive means for detecting relative changes in myocardial elasticity during the cardiac cycle [9-11]. This method employs low-frequency shear waves induced in the heart by an external harmonic vibrator and measures the wave amplitude variation (WAV). This WAV results from changes in myocardial stiffness during the cardiac cycle. A linear elastic model was used to transform WAV to elasticity ratios. To further exploit cardiac MRE, a linear pressure-stiffness model was developed to utilize WAV-MRE for noninvasive measurement of left ventricular (LV) pressure ratios. A study of eight healthy volunteers showed good correlation between relative pressure values derived by MRE and data available from the literature [10]. While this correlation confirmed the validity of the assumptions made for WAV-MRE [9] in principle, MRE was not correlated with LV pressure because invasive examination of pressure-time function is precluded in healthy volunteers.

Therefore, we here present a study investigating the correlation between wave amplitudes in MRE and LV pressure determined by catheter measurement in pigs. As both experimental measures are expected to vary widely due to individual heart morphology and function three different animals showing significant differences in weight, age and physiologic cardiac parameters were investigated. Our intention was to show the evolution of geometry, pressure and shear stimulus of the heart under different physiological conditions applying neither elastodynamic assumptions nor geometrical models. WAV-time functions are deduced from raw wave-phase data by an algorithm introduced here that identifies regions of contracting tissue. Then, the correlation of WAV-MRE to LV pressure and geometry is analyzed by linear regression.

## Methods

### Animals and Anesthesia

All experiments were approved by the responsible authority. Three pigs (mini LEWE) with a body weight of 25-53 kg were used in this study. Before the experiments, anesthesia was induced by intramuscular injection of 15 mg/kg of ketamine hydrochloride (Ketamin^® ^100 mg/ml; Pfizer, Karlsruhe, Germany), 0.1 mg/kg droperidol (Droperidol 2.5 mg/ml; Hospira, Lake Forest, USA), and 0.2 mg/kg midazolam hydrochloride (Dormicum^®^; Hoffmann-La Roche AG, Grenzach-Whylen, Germany). To deepen anesthesia for intubation, propofol (propofol 1%, Fresenius Kabi, Bad Hombug, Germany) was administered into a lateral ear vein. After the anesthetics took effect, a 6.0-7.0 mm endotracheal tube (Mallinckrodt Laboratories, Athlon, Ireland) was inserted into the trachea to maintain anesthesia with a mixture of 2-3 vol% isoflurane (Forene; Abbott, Wiesbaden, Germany) and medical oxygen. Anesthesia was maintained using an electronic system for controlling ventilation and anesthesia (A.D.S. 1000, engler engineering corporation, Florida, USA). Directly prior to catheterization, 200 IU/kg heparin sodium (Liquemin^® ^N 25.000, Hoffmann-La Roche AG, Grenzach-Wyhlen, Germany) was administered as an intravenous bolus. To maintain identical heart rates for LV pressure measurement and MRE experiments, the pigs received a continuous intravenous infusion of metoprolol tartrate (Beloc i.v., AstraZeneca GmbH, Wedel, Germany) at a rate of 50 ml/h.

### Left Ventricular Pressure Measurement

The tip manometer (model PC-380, Millar Systems, Houston, Texas) was advanced into the heart via the right carotid artery using a guide-wire under fluoroscopic control. Tip manometer catheters were adjusted to atmospheric pressure prior to measurement. Pressure-time functions were stored together with electrocardiograph (ECG) signals in a digital oscilloscope (200 Hz sampling rate, 12 bit A/D converter). The time axis of the manometer data was subsequently shifted to match the R-waves of the MRE experiments using the ECG recordings.

### Cardiac MRE

Details of the acquisition protocol are described in [10,11]. Standard short-axis cine SSFP images served to calculate LV volume and function in each animal. Directly after catheter intervention and recording of the LV pressure time course, MRE experiments were run in a clinical 1.5 T scanner (Magnetom Sonata, Siemens AG, Germany) using a standard 12-element body phased-array coil. A remote mechanical driver [12] was used to vibrate the left lateral chest of the animals at 48.3 Hz. An ECG-triggered, motion-sensitized, spoiled gradient echo sequence was run to acquire wave images with a frame rate of 193 Hz. (repetition time, TR: 5.18 ms; echo time, TE: 1.38 ms; flip angle, α: 15°, length of motion-encoding gradient (MEG): 2 ms; MEG amplitudes: 33 mT/m, 25 mT/m and 21 mT/m along the directions of read-out, phase-encoding and slice selection, respectively). Other imaging parameters were 200 × 400 mm field of view (FOV), 6 mm slice thickness in a short-axis view, and 96 × 128 matrix size. Segmented k-space acquisition was applied with one phase-encoding step per ECG trigger. Generalized autocalibrating partially parallel acquisition (GRAPPA; factor 2) was used. Two hundred eighty wave images were acquired in approximately 1 minute during ventilation stop. Left ventricular diameter was calculated from the magnitude images of the MRE sequence. Therefore the magnitude images of all three spatial directions are superimposed using custom-made software run on MATLAB (Version 7.0.4, The MathWork Inc.) and left ventricular diameter was measured repeatedly by a physician experienced in cardiovascular MR.

### Data Evaluation

The principles of evaluating WAV-MRE data have been described previously [11]. A crucial processing step was the calculation of the correlation of the encoded cardiac motion with a complex oscillation of frequency of the externally induced shear oscillations. The magnitude of this complex correlation yielded shear wave amplitudes while intrinsic heart motion was suppressed. As proposed in [11] and revisited in the Appendix, a correlation function was employed to isolate regions of contracting tissue from those of noncontracting tissue by using a diastolic gating function *S *(*S *= 1 for times attributed to diastole and otherwise *S *= 0). Experimentally, *S*(*t*) was manually selected once in each animal from the alteration of the wave amplitudes. Correlation of *S*(*t*) with wave amplitudes *U*(*t*) resulted in the time-independent map of WAV, denoted by  indicating regions of contracting tissue. In the Appendix, this approach of estimating the region of contracting tissue is further elaborated and essential equations are given. The standard process of quantifying the WAV effect involved three steps: i) manual segmentation of LV boundaries during systole (giving a coarse region of interest, *ROI*); ii) calculation of correlations of wave amplitudes *U*(*t*) with *S*, yielding  using Eq.A3 in the appendix; and iii) automatic refinement of the *ROI *depending on the correlation intensity given by  using the following equation:

(1)

*x *is the location of a point within the *ROI *and *ζ *is an empirical factor between 0 and 1 that determines the optimal spatial extension of the *ROI *based on the point of maximum amplitude variation, . In the present study the WAV effect was evaluated for *ζ *= 0, 0.1, .1.0, and *ζ *= 0.9 was chosen after further discussion.

## Results

**Figure 1:**
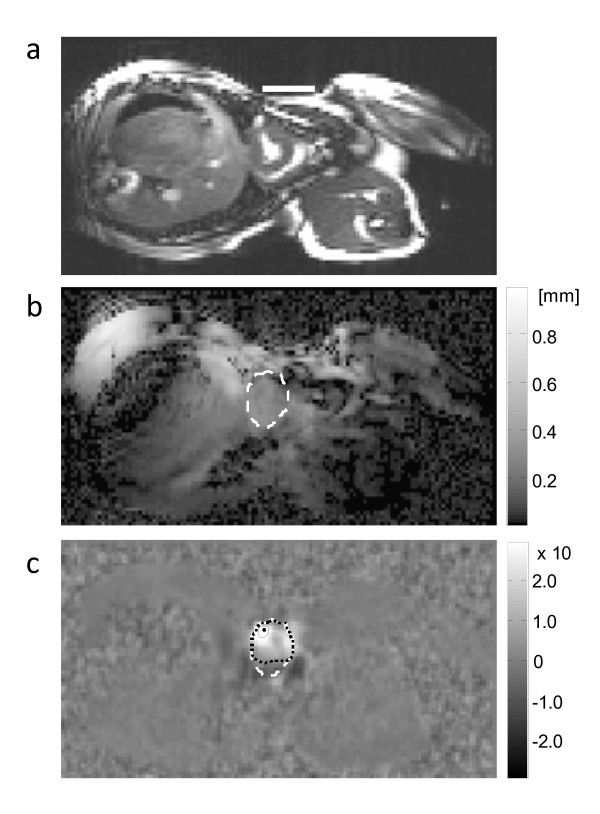
**MRE of a pig heart in the short-axis view**. a) Standard cine MR in a systolic heart phase. The white line on the lateral chest wall of the pig marks the connection plate of the mechanical driver. b) Resultant MRE wave amplitudes of 48.3 Hz drive frequency combining all three spatial wave components (the dashed line indicates the systolic LV circumference obtained by manual segmentation). c) Correlation map showing the regions where wave amplitudes vary in synchrony with the heart beat. Dashed, dotted, and solid lines symbolize systolic LV boundaries, and ROIs corresponding to *ζ *= 0 and *ζ *= 0.9, respectively. The single dot indicates *ζ *= 1, i.e. maximum .

**Figure 2:**
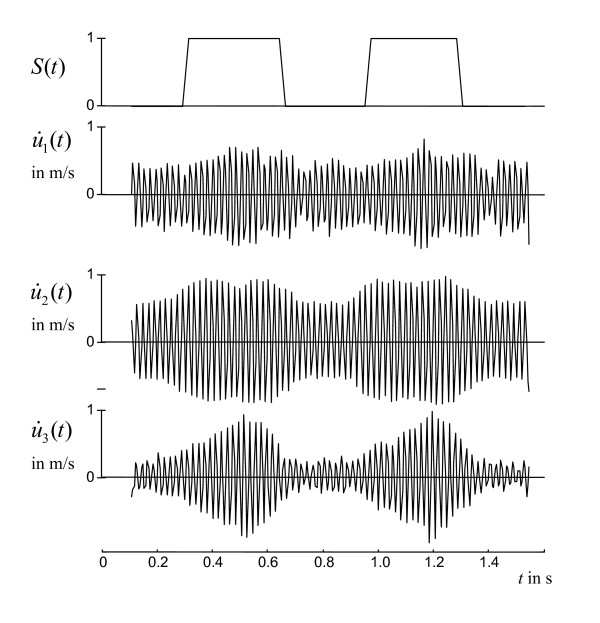
**48.3 Hz vibration components *u*_*i *_acquired in the same animal as shown in figure 1 (1 = read out direction, 2 = phase encoding direction and 3 = direction through the image plane)**. The used *ROI *was derived by *ζ *= 0.9. Shown are the temporal derivatives of spatially averaged oscillations representing widely flow-suppressed phase oscillations [11].

**Figure 3:**
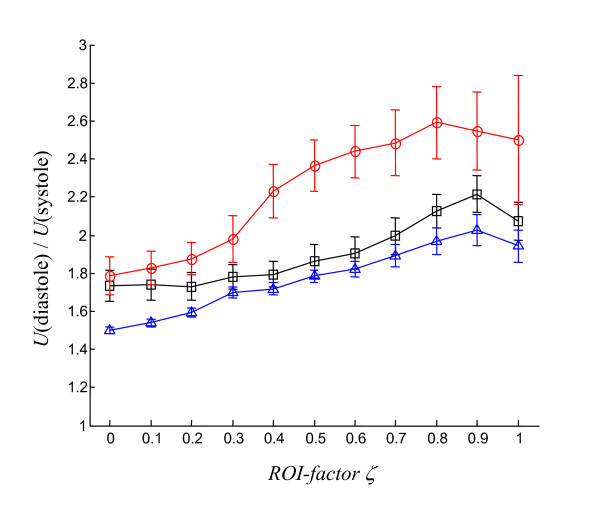
**Shear wave amplitudes *U*(*t*) of the three pigs averaged within different *ROI*s determined by *ζ *(Eq. 1)**. *U*(*diastole*)/*U*(*systole*): temporal mean of the ten highest/lowest values of *U*(*t*). Each animal is represented by a different symbol (triangle #1; square #2; open circle #3)."

**Figure 4:**
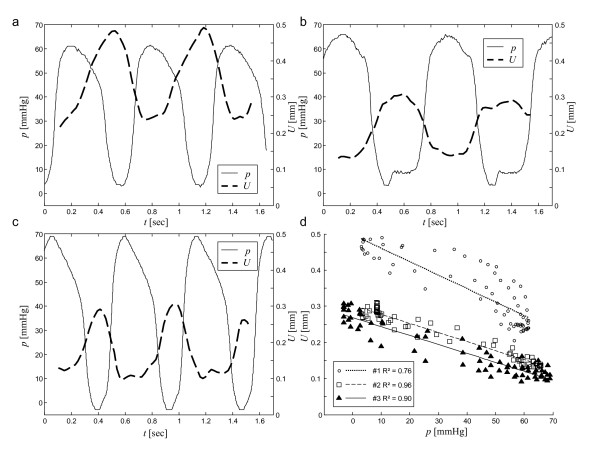
**Variation of wave amplitudes *U*(*t*) observed by MRE compared with invasively measured LV pressure *p*(*t*) in three pigs (a, b, and c, corresponding to animals 1, 2 and 3)**. The animals investigated had different heart rates and pressure levels, giving rise to different relative wave amplitude changes and amplitude offsets in MRE. Figure d shows the linear regression of *U(p) *in all pigs.

**Figure 5:**
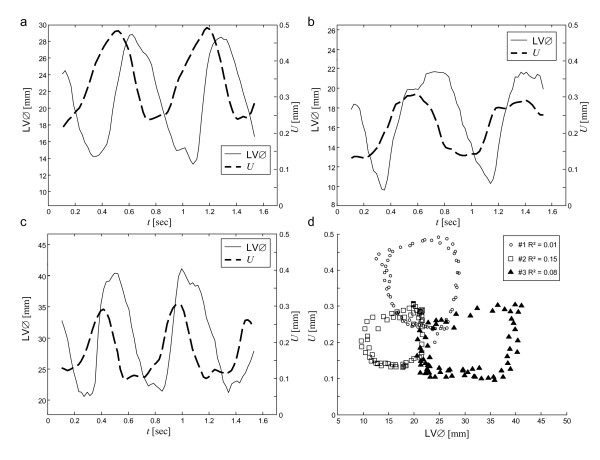
**Amplitudes *U*(*t*) and LV diameter (LV∅) determined from the MRE magnitude images along the apical short cardiac axis**. (a, b, and c, corresponding to animals 1, 2 and 3). Figure d shows the change in wave amplitudes over LV diameters, indicating that there is no linear correlation between these measures. The characteristic shape of the loops is well known from pressure-volume cycles [14,10].

Individual physiological data are summarized in Table 1. Almost uniform shear wave penetration of thoracic and abdominal tissue was achieved using 48-Hz drive frequency. The magnitude of the shear wave amplitudes averaged over the entire cardiac cycle appears not to be significantly damped within the heart (Fig. 1). Spatially averaged oscillation components are shown in Figure 2. Furthermore, a manually selected gating function *S *is shown, which was used for calculating the correlation map  (see Eq.A3) in animal #1 (see Fig. 1c). Figure 1c demonstrates that most of the image intensity  > 0 is located within the systolic boundaries of the left ventricle. Within that region, the modulation of wave amplitudes during the measurement period correlates with the diastolic gating function *S*. Two *ROI *boundaries within the systolic left ventricle are demarcated, corresponding to  > 0 and . The region of  > 0 encircles a lower mean correlation signal than regions of  with *ζ *> 0 since the former region may include blood or non-LV tissue pockets. The higher the correlation signal in a specific region of  the higher the underlying ratio of wave amplitudes, *U*(diastole)/*U*(systole). In Figure 3 this ratio is plotted against *ζ *for all three animals. It is clearly visible that in all three experiments the amplitude ratio increases with *ζ*, reaching a maximum at approximately *ζ *= 0.9, which is further used for the quantification of our MRE data. From *ζ *= 0 to *ζ *= 0.9, ROI sizes decreased from 898 to 65 mm^2 ^in animal #1, from 794 to 123 mm^2 ^in animal #2, and from 787 to 189 mm^2 ^in animal #3. Figure 4 shows the time dependence of both MRE wave amplitudes *U*(*t*) and invasively measured LV pressure. It is clearly visible that *U*(*t*) alternates in synchrony with the pressure inside the left ventricle, confirming the validity of the correlation of LV pressure and MRE-WAV effect proposed in [10]. This correlation is further illustrated in Fig. 4d, where the linear regression of *U*(*p*) of all three animals is plotted and the resulting R^2 ^values are given (mean R^2 ^= 0.87). The least correlation (R^2 ^= 0.76) was found for animal #1, which displayed the lowest WAV effect, given by *U*(diastole)/*U*(systole) = 2.03 ± 0.08. The other two animals show a higher correlation (R^2 ^= 0.96 and R^2 ^= 0.90). The increase in amplitude ratios from animal #1 to animal #3 is consistent with the increase in pressure values given in Table 1. The mean WAV ratio in all three animals was 2.35 ± 0.4. Figure 5 shows the comparison of MRE wave amplitudes and LV diameters, which were manually determined from MRE magnitude images. It is clearly visible that wave amplitudes change ahead of LV geometry similar to the well-known phases of isovolumetric contraction (IVC) and isovolumetric relaxation (IVR). Estimations of the delay between WAV and changes in LV diameter during IVC and IVR are given in table 1. IVC was 102 ± 58 ms, which is shorter than IVR (175 ± 40 ms) and is consistent with findings of cardiac MRE in humans [10]. IVC times appear to be related to *U*(systole) and *U*(diastole)/*U*(systole), which was not observed for IVR. The occurrence of isovolumetric phases is seen with *U*-LV diameter cycles as shown in figure 5d. Low correlation coefficients of R^2 ^≤ 0.15 (mean R^2 ^= 0.08) reflect the asynchronous alteration of WAV and heart geometry.

**Table 1:** Physiological cardiac parameters.

**Animal #**	**Weight** **[kg]**	**Heart rate** **[bpm]**	**LV ejection fraction [%]**	**LV end diastolic volume [ml]**	**p_peripheral_** **[mm Hg]**	**Δp_invasive_** **[mm Hg]**	***U*(systole)** **[mm]**	***U*(diastole)/*U*(systole)**	***IVC*** **[ms]**	***IVR*** **[ms]**
1	53	77	51	41	66	60	0.237 (0.003)	2.03 (0.08)	169 (26.5)	176 (19.0)

2	23	61	54	35	69	65	0.136 (0.002)	2.22 (0.10)	79.5 (8.5)	214 (23.0)

3	25	93	55	70	75	73	0.104 (0.002)	2.79 (0.15)	59.5 (14.5)	134 (9.0)

The wave amplitudes *U*(systole) and *U*(diastole) were determined for *ζ *= 0.9 and averaged over the ten lowest and ten highest data points of *U*(*t*), respectively.

Delays of isovolumetric contraction (IVC) and isovolumetric relaxation (IVR) were ten times manually selected in a range of 30 to 70% of the maximum of *U*(*t*) and the change of left ventricular diameter. Standard deviations are given in brackets.

## Discussion

The feasibility of amplitude-sensitive MRE for measuring tissue-elastic properties was demonstrated by phantom experiments for plane interfaces in [11] and for cylindrical inclusions in [13]. This study compares, for the first time, MRE and cardiac pressure data. The synchronization of the MRE-derived wave amplitude alteration with invasively measured pressure changes corroborates the validity of a central assumption made previously in cardiac MRE: The change in shear wave amplitudes over the cardiac cycle is caused by elasticity changes in the myocardium and not driven by geometrical effects. Provided that stiffening and relaxation of the heart wall cause an immediate change in pressure, our results underscore that WAV-MRE is sensitive to LV pressure dynamics rather than to cardiac motion. An instantaneous response of LV pressure to myocardial contraction is well known from animal studies [14-16]. The comparison of WAV-MRE with LV pressure provides the missing link towards noninvasive assessment of cardiac pressure-volume variations.

The present study was focused on the principal relationship between MRE signal and invasively measured pressure. The most important result of this study is that there is a clear distinction between the coherences of WAV pressure and WAV geometry functions: While wave amplitudes vary (reciprocally) in synchrony with pressure changes (fig. 4) there is a clear delay between MRE and LV-diameter (fig. 5). Despite the small number of animals investigated this result is significant since the physiological differences seen in our vivo model covers a broad spectrum of pig heart physiology. Most noticeable, animal #1 was about twice the weight of the other two animals. However, Table 1 does not indicate a correlation between weight and heart rate, ejection fraction or LV volume. Instead, the increase in pressure parameters from animal #1 to #3 is reflected in the *U*(dia)/*U*(sys). Thus, the distinct offset of absolute wave amplitudes seen in animal #1 (Table 1, column *U*(systole)) is most probably attributable to different wave conditions resulting from the greater body extent imposing different wave conditions and not to effects of cardiac physiology. Yet, it is an encouraging result that this offset appears not to influence relative ratios *U*(diastole)/*U*(systole), which increase monotonically from animal #1 to #3. This observation suggests that WAV-MRE is a suitable technique for determining relative changes in left ventricular pressure. The diagnostic value of WAV-MRE has to be addressed in future studies. Additionally, ex vivo cardiac models (including tissue models) are needed for clarifying principles of shear waves in tissue exhibiting a wide variation in elasticity. In this respect, it is important to note that despite the good agreement in the timing of WAV and LV pressure changes, their dynamics are clearly different (see Figure 4). Differences in slopes and level times may result from nonlinear elastic effects in myocardial tissue. Moreover, the site from which LV pressure data are sampled relative to the position where the WAV effect is quantified may impact the coherence of both measures. The proposed method for selection of the appropriate *ROI *is a semi-automatic way to identify the maximum ratio *U*(diastole)/*U*(systole). It was found that the greatest WAV effect occurs in regions well exposed to shear vibrations in the vicinity of the mechanical transducer. Provided that the linear elastic model of Eq.A2 applies, the degree of amplitude variations at *ζ *= 0.9 indicates relative pressure ratios from 17 (animal #1) to 60 (animal #3). This large range is assumed to result from a forth-power law in Eq.A2 rather than reflecting physiological variability.

In summary, mechanical stimulation of the heart by low-frequency shear vibrations enables measurement of time-dependent ratios of wave amplitudes using cardiac MRE. We demonstrated that such ratios correlate with ventricular pressure and hence provide a noninvasive measure of ventricular pressure ratios. Our results suggest that the degree of wave amplitude alterations agrees with individual differences in cardiac physiology in terms of LV pressure variation.

## Competing interests

The authors declare that they have no competing interests.

## Authors' contributions

TE, IS and BH designed the study. ML, NK, JS and TE carried out the animal experiments. IS, TE and JB evaluated data and performed the statistical analysis. IS, TE and AS wrote the manuscript. All authors read and approved the final manuscript.

## Appendix

Shear wave amplitudes *U *are determined from phase oscillations along all three Cartesian components (*U*_*i*_, *i *∈ {1,2,3}) acquired in three successive experiments with different directions of the motion-encoding gradients:

(A1)

A linear elastic model can be used to relate wave amplitude changes at two different time points, *t*_1 _and *t*_2_, during the cardiac cycle to myocardial stiffness changes [10,11]:

(A2)

It is important in cardiac MRE to identify regions where *U*(*t*) changes during the cardiac cycle to identify regions of contracting myocardium and to segment them from blood and surrounding noncontracting tissues. In the following, *U*_*n *_corresponding to discrete time increments *n *is analyzed by correlation , which is defined as follows:

(A3)

Here, *N *is the number of points in *U *(35 in our experiments), and *S *is a step function defined on the basis of the duration of systole and diastole.  becomes nonzero for points within contracting tissues and zero for points corresponding to noncontracting tissues. Therefore  is an appropriate function for threshold-based segmentation of regions of different WAV characteristics. For example, based on the definition of *S*, the highest ratio *U*(diastole)/*U*(systole) is theoretically determined from the region of maximum . Such a region tends to be small, however, and because noise is present, averaging over such a small region may lead to inaccurate results. In principle, the proper region of interest should be determined based on the two competing factors of high  values and large region size. For this purpose, we define the *ROI *within the heart such that its size and position are linked to  by the factor *ζ *(Eq.1). Figure 1 illustrates that *ζ *= 1 results in a small *ROI *at maximum  whereas *ζ *< 1 values cause the *ROI *to increase in size, reducing the effect of noise in the spatially averaged function *U*(*t*).
